# Generation of flower high-order Poincaré sphere laser beams from a spatial light modulator

**DOI:** 10.1038/srep39657

**Published:** 2016-12-21

**Authors:** T. H. Lu, T. D. Huang, J. G. Wang, L. W. Wang, R. R. Alfano

**Affiliations:** 1 Department of Physics, National Taiwan Normal University, 88 Tingchou Road, Sec. 4, Taipei 11677, Taiwan; 2 Institute for Ultrafast Spectroscopy and Lasers, Physics Department, The City College of New York of the City University of New York, New York, NY 10031, USA

## Abstract

We propose and experimentally demonstrate a new complex laser beam with inhomogeneous polarization distributions mapping onto high-order Poincaré spheres (HOPSs). The complex laser mode is achieved by superposition of Laguerre-Gaussian modes and manifests exotic flower-like localization on intensity and phase profiles. A simple optical system is used to generate a polarization-variant distribution on the complex laser mode by superposition of orthogonal circular polarizations with opposite topological charges. Numerical analyses of the polarization distribution are consistent with the experimental results. The novel flower HOPS beams can act as a new light source for photonic applications.

Over the past few years, structured light with space-variant polarization and phase distribution has attracted considerable interest because of the broad applications of photonic quantum information, quantum entanglement, and quantum cryptography^1^,^2^,^3^,^4^,^5^. Several approaches have been proposed for generating structured light, such as laser resonators, q-plates, holograms, and spatial light modulators (SLMs)^6^,^7^,^8^,^9^,^10^,^11^,^12^,^13^,^14^,^15^. SLMs have been employed for generating structured beams to manifest optical angular momentum and optical vortices, especially for Laguerre-Gaussian (LG) modes^16^,^17^,^18^,^19^. A spatially inhomogeneous polarization state of LG modes can be described as a point on the surface of a high-order Poincaré sphere (HOPS) that can be used to represent the higher-order modes of optical fibers, vector beams, and the Pancharatnam-Berry geometry phase for higher-order states of polarization^20^,^21^. Generating controllable complex structured beams with inhomogeneous polarization states mapping onto HOPSs can pave the way for exploring advanced applications^6^,^22^,^23^,^24^.

In this paper, we experimentally produced a new complex laser mode from the superposition of multiple LG modes mapped on HOPSs. We observed that the localization of the superposed modes displayed a new type of flower-like patterns. Passing through simple optical elements enabled light to assume modes with flower-like structures and space-variant distribution of polarization.

## Results

### Generating structured beams from a SLM

**Figure 1:**
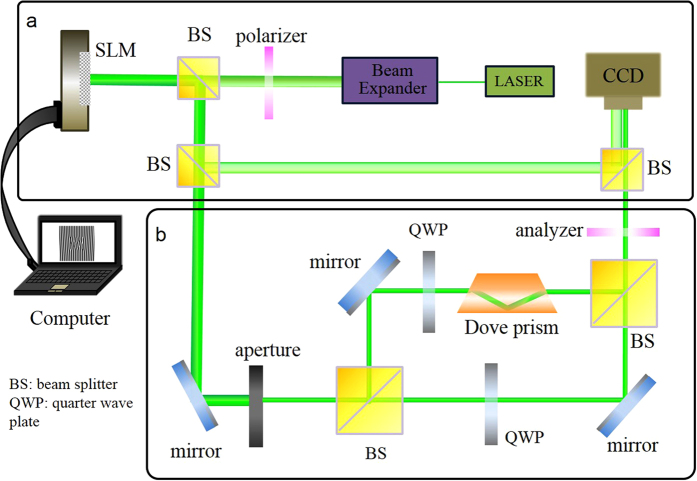
Experimental setup: (**a**) Generating pure LG modes and flower modes from a phase-only spatial light modulator. (**b**) Generating flower modes with inhomogeneous polarization distributions.

The experimental setup is shown in Fig. 1. Light emitted from a diode-pumped solid state laser was collimated and expanded through a beam expander before it was sent onto a reflective phase-only liquid crystal on silicon SLM. The experimental detail is given in the method summary. Grating phase patterns of desired modes were applied to the liquid crystal display of the SLM. The phase of the incident plane waves was modulated and leads to complex structured beams according to the grating phase. These complex structured beams were divided into two parts by a beam splitter. One part was reflected into a charge-coupled device camera to record its intensity. The other part, shown in Fig. 1b, passed through optical components for coding on the HOPS. An adjustable aperture was used to select the first order of the reflecting patterns diffracted from the grating phase displayed on the SLM. Two orthogonal quarter-wave plates were utilized to generate circularly polarized beams with opposite handedness, and a dove prism in one arm converted the LG mode with an azimuthal index 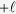 to the opposite azimuthal index − 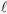.

Consequently, the right-handed circularly polarized LG mode of index 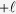 combined with the left-handed circularly polarized LG mode of index − 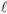. A polarizer was placed before the CCD camera to analyze the polarization state of the structured beam. Vector-structured beams of single and multiple LG modes can be generated and analyzed by this simple experimental setup.

### Intensities and phase distributions of the flower modes

**Figure 2:**
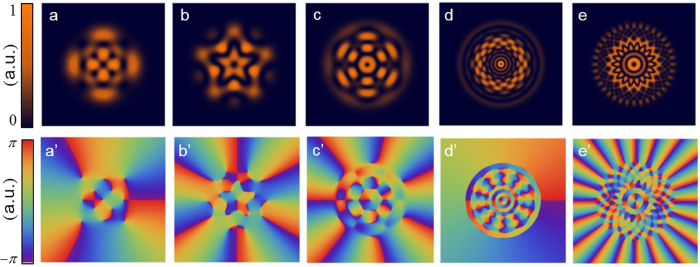
(**a**)–(**e**) Numerical results of the superposed LG modes represented by Eq. (1). (**a**) *LG*(1, 3, 0, −4, 1), (**b**) *LG*(1, 4, 0, −5, 2), (**c**) *LG*(2, 1, 0, 6, 1), (**d**) *LG*(0, 10, 9, −11, 1), (**e**) *LG*(3, 16, 0, −18, 2). (**a**’)–(**e**’) Phase distribution corresponding to (**a**)–(**e**).

**Figure 3:**
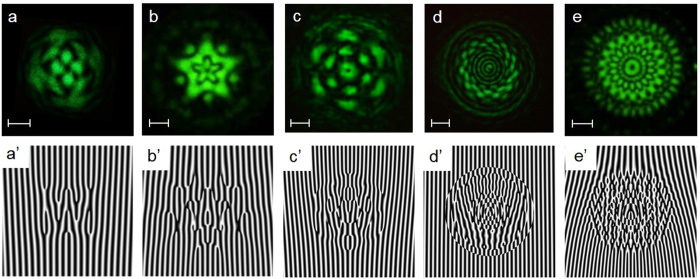
(**a**)–(**e**) Experimental results corresponding to Fig. 2(a)–(e). The scale bars represent a length of 1 mm. The results were measured at a distance of 108 cm from the SLM. Numerical grating phases display on the SLM to generate flower laser beams shown in (**a**)–(**e**).

The wave function of LG mode, 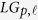, with a radial index *p* and azimuthal index 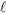 defines the basis of the flower-structured mode. The superposed LG mode can be represented as


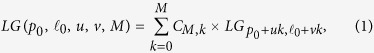


where 
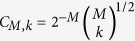
 is the weighting coefficient^8^. Figure 2a–e show the numerical results of flower modes. The characteristic number of petals depends on the interval of azimuthal index *v* of the superposed LG mode. The interval of the radial index *u* of the superposed LG mode determines the complexity of the radial symmetry. Figure 2a’–e’ illustrate the phase distributions corresponding to the intensity distributions presented in Fig. 2a–e. The phase singularities localized on the points of zero intensity also construct complex patterns related to the intensity profiles. Different from a conventional single LG mode which has uniform azimuthal intensity, the superposed LG modes cause an exotic flower pattern formation. Furthermore, the corresponding phase singularities also constitute flower phase patterns. Figure 3a–e depict the experimental intensity distributions generated from the arrangement shown in Fig. 1a. To generate the complex flower beams, the grating phase patterns were used to display on the SLM for coding the phase information onto the incident plane wave. Figure 3a’–e’ illustrate the grating phase patterns corresponding to the intensity distribution presented in Fig. 3a–e. High-resolution SLM makes it possible to distinguish the precise difference of the grating phase patterns and generate various complex flower beams. The size of the complex flower beam is proportional to the spot size of the laser beam incident into the SLM and the order of the superposed LG mode. The divergent angle of the flower beams showed in Fig. 3 is less than 0.2 mrad. The physical scale of the structured beams is manipulable under the different optical parameters of the setup.

### Flower HOPS laser beams

**Figure 4:**
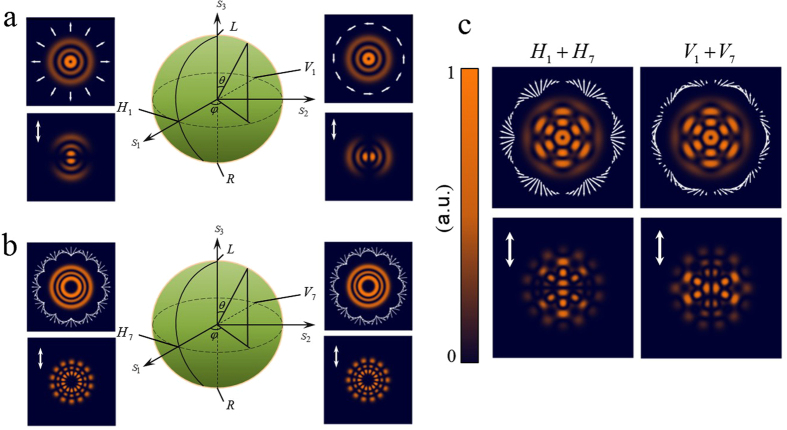
(**a**) Numerical intensity represented on a HOPS for the *LG*_2,1_ mode. Horizontal and vertical bases, H_1_ = ψ_1_(π/2, 0) and V_1_ = ψ_1_(π/2, π), are the radial polarized state and azimuthal polarized state. The bottom figures show the numerical transmitted intensities from a vertical linear polarizer. The poles represent orthogonal left-handed and right-handed circularly polarized bases. (**b**) Numerical intensity and polarization distribution represented on a HOPS for the *LG*_2,7_ mode. There are seven nodal lines on the transmitted intensities from a vertical linear polarizer. (**c**) First row: Numerical intensity and polarization distribution of the superposed LG mode *LG*(2, 1, 0, 6, 1) in terms of *H*_1_ + *H*_7_ and *V*_1_ + *V*_7_ states. Second row: Numerical transmitted intensities from a vertical linear polarizer.

For arbitrary points on the HOPS shown in Fig. 4a, the state of polarization 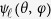 can be represented as


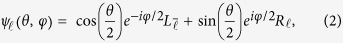


where *θ* is the polar angle, and *φ* is the azimuthal angle in spherical coordinates. 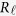 and 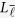 are the right-handed and left-handed circular polarization basis such that 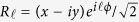 and 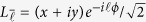. The factor 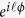 is the vortex phase associated with the LG mode of azimuthal index 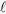 possessing orbital angular momentum. Any desired vector beam on the HOPS can be experimentally achieved, as was shown in a previous study^19^. The state of polarization 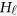 and 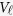 can be represented as


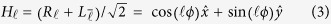


and


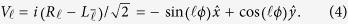


Figure 4a shows the numerical results of the intensity distribution and the corresponding polarization states 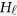 and 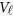 of *LG*_2,1_. For the first-order (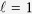) LG mode, the state 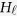 indicates radial polarization, and 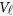 indicates azimuthal polarization. The distribution of the polarization state depends only on the azimuthal index 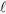 of the LG mode regardless the radial index *p*. Figure 4b illustrates the numerical results of the intensity distribution and the corresponding polarization states 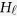 and 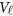 of *LG*_2,7_. The variation of polarization states along azimuthal direction becomes more frequent according to the azimuthal index. The polarization states 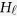 and 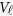 bring about 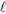 nodal lines on the transmitted intensity from a linear polarizer oriented in the vertical direction. However, for a flower mode arising from the superposition of multiple LG modes, the polarization state embedded in the interference of multiple LG modes is rearranged. Figure 4c shows the numerical intensity and the superposed polarization state of the flower mode. The polarization state of the flower mode is more complex than the polarization state of a single LG mode.

**Figure 5:**
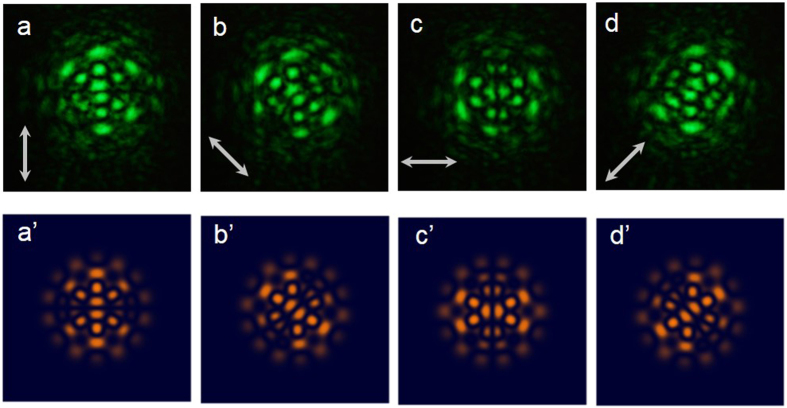
(**a**)–(**d**) Polarization-resolved patterns according to the pattern in Fig. 3(c) for different angles of the analyzer shown in Fig. 1(b). (**a**’)–(**d**’) Numerical results for (**a**)–(**d**).

The experimental setup shown in Fig. 1 was designed to generate inhomogeneous polarization flower modes arising from the superposition of multiple LG modes mapping to the polarization state 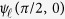, which is the point 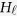. The combination of left-handed and right-handed circularly polarized flower modes with opposite topological charges can be demonstrated. The intensity of the mode combination is unchanged because of the orthogonal polarized states of the two flower modes. Notably, the flower mode combination is transformed to an inhomogeneous polarization flower mode. Figure 5a–d depict the experimental polarization-resolved patterns corresponding to the flower mode shown in Fig. 3c. Obviously, the center circle is radially polarized, which does not follow the polarization distribution presented in Fig. 4c. The spatial overlap of the two bases, *LG*_2,1_ and *LG*_2,7_, dominates the polarization distribution of the flower mode. The different radial intensities of the two bases lead to different polarization distributions along radial direction of the vector flower mode. Different from the radial-independent polarization distribution for conventional HOPS beams, the polarization distribution of the flower HOPS laser beam is radially dependent.

**Figure 6:**
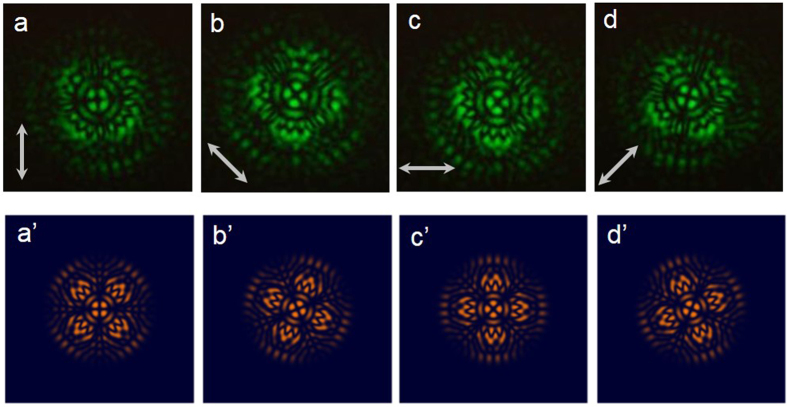
(**a**)–(**d**) Polarization-resolved patterns according to the pattern in Fig. 3(e) for different angles of the analyzer shown in Fig. 1(b). (**a**’)–(**d**’) Numerical results for (**a**)–(**d**).

The analyses of numerical intensities under the different polarization directions that are shown in Fig. 5a’–d’ help to explain the experimental polarization-resolved patterns shown in Fig. 5a–d. Figure 6a–d illustrate the experimental polarization-resolved patterns corresponding to the flower mode shown in Fig. 3e. The polarization of the center circle has four-fold symmetry because one of the azimuthal indexes 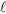 of the bases is 2. The polarization distribution of the flower mode is more complex than conventional HOPS beams. The numerical results shown in Fig. 6a’–d’ also have notable agreement with the experimental results. The flower modes with complex inhomogeneous polarization mapping on HOPSs can be generated systematically. Manipulation of the polarization state mapping on arbitrary points on HOPS can be achieved by adding other optical components in our experimental setup for future work.

## Discussion

Conventional HOPS beams generated from various optical systems as laser cavities, SLM, hologram, and q-plates are all related to the optical modes of topological charge 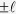 and independent of radial distribution of intensity and polarization. A great deal of effort has been made on HOPS beams. What seems to be lacking, however, is the spatial degree of freedom along radial direction for HOPS beams. We experimentally and theoretically demonstrate novel flower HOPS laser beams with complex polarization distribution along both azimuthal and radial directions. These exotic flower HOPS laser beams can provide applications including vector mode multiplexing, optical manipulation, and quantum communication.

In conclusion, we have used an optical system, including a phase-only SLM, to generate a new form of laser beam sculpting. These complex structured modes can be decomposed into LG modes mapping to HOPSs. The structures of the flower HOPS beams are highly stable and can be manipulated systematically by changing the grating phase displayed on the SLM. All the experimental polarization-resolved patterns have been adequately analyzed with the superposed LG modes. The results show a notable intensity and polarization distribution and provide useful insights for potential photonic applications for information and communications.

## Method summary

A diode-pumped solid state laser with wavelength of 532 nm and output power of 20 mW was expanded by a 6× beam expander and collimated onto a reflective phase-only SLM of 1920 × 1080 pixels with pixel pitch of 8 μm. The grating phase pattern of a desired mode was displayed onto the SLM. The laser beam with a beam size of 8 mm diameter was incident on the SLM and reflected diffraction pattern from the SLM. The intensity profiles can be reflected to a CCD camera by the two beam splitters shown in Fig. 1a. To modulate the polarization states of the structured beams, two orthogonally oriented quarter-wave plates were used to transform linearly polarized beam into two circularly polarized beams with opposite handedness shown in Fig. 1b. The adjustable aperture selected the first-order diffraction pattern for further analysis. Dove prism was used to change the handedness of LG modes. A Mach-Zehnder interferometer was built for generating sculpted flower HOPS laser beams with inhomogeneous polarization.

## Additional Information

**How to cite this article**: Lu, T. H. *et al*. Generation of flower high-order Poincaré sphere laser beams from a spatial light modulator. *Sci. Rep.* **6**, 39657; doi: 10.1038/srep39657 (2016).

**Publisher's note:** Springer Nature remains neutral with regard to jurisdictional claims in published maps and institutional affiliations.
